# Independent Anatomic Double‐Bundle Anterolateral Ligament Reconstruction Using a Pedicled Gracilis Graft During Anterior Cruciate Ligament Reconstruction

**DOI:** 10.1002/atn2.70044

**Published:** 2026-09-25

**Authors:** Ibrahim Marzouki, Anthony Viste, Thomas Neri

**Affiliations:** ^1^ Chirurgie Orthopédique et Traumatologique Hospices Civils de Lyon, Hôpital Lyon Sud Lyon France; ^2^ University Lyon, Claude Bernard Lyon Villeurbanne France; ^3^ LBMC UMR_T9406 University, Gustave Eiffel University, IFSTTAR Lyon France

## Abstract

Recent studies on the anatomy and biomechanics of the anterolateral ligament (ALL) have highlighted its fundamental role in rotational control of the knee. These findings have led to the integration of its reconstruction into anterior cruciate ligament (ACL) reconstruction strategies, in order to improve management of residual pivot shift and optimize knee stability. This article describes a minimally invasive technique for ALL reconstruction, independent of the ACL. The procedure is based on the use of a pedicled autograft of the gracilis tendon. The ALL reconstruction follows a pedicled approach, ensuring optimal anatomical and biomechanical restoration. The graft is first directed to the distal insertion of the native ALL on the tibia, equidistant between Gerdy's tubercle and the head of the fibula, then routed to its femoral insertion, passing deep under the iliotibial band, next back on itself before being redirected to its initial point of emergence in the tibial tunnel. After fixation at the femoral level, tibial anchoring is performed in full extension to optimize isometry.

## Graphical abstract

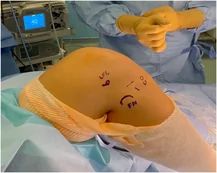

**VIDEO 1:** Overview and Technical demonstration of independent anatomic ALL reconstruction using a pedicled Gracilis graft. Video content can be viewed at https://doi.org/10.1002/atn2.70044.

Recent anatomical and biomechanical studies have focused and confirmed the function of the anterolateral ligament (ALL) in knee rotational stability,^1^, ^2^, ^3^ leading to the development of several techniques for anatomic reconstruction.^4^

The following technique is our primary procedure for simultaneous anterior cruciate ligament (ACL) and ALL reconstruction using tripled semitendinosus (ST) graft for ACL and isolate anatomic reconstruction with gracilis graft folded in 2 strands. A pedicled reconstruction technique is employed for both ligaments.

## SURGICAL TECHNIQUE

### Patient Positioning and Anatomic Landmarks

The patient was positioned supine, with a lateral wedge positioned at tourniquet level. Two‐foot roll were placed flush with the table to position the knee at 90° flexion (working position) and 30° flexion (position for fixing the ACL and ALL). This set‐up enabled extension, flexion, and rotation of the knee during the surgical procedure without constraints while avoiding hip‐induced external rotation.

Bony landmarks were drawn on the lateral aspect of the knee: lateral epicondyle, Gerdy's tubercle, fibular head, and joint space (Figure 1).

**FIGURE 1:**
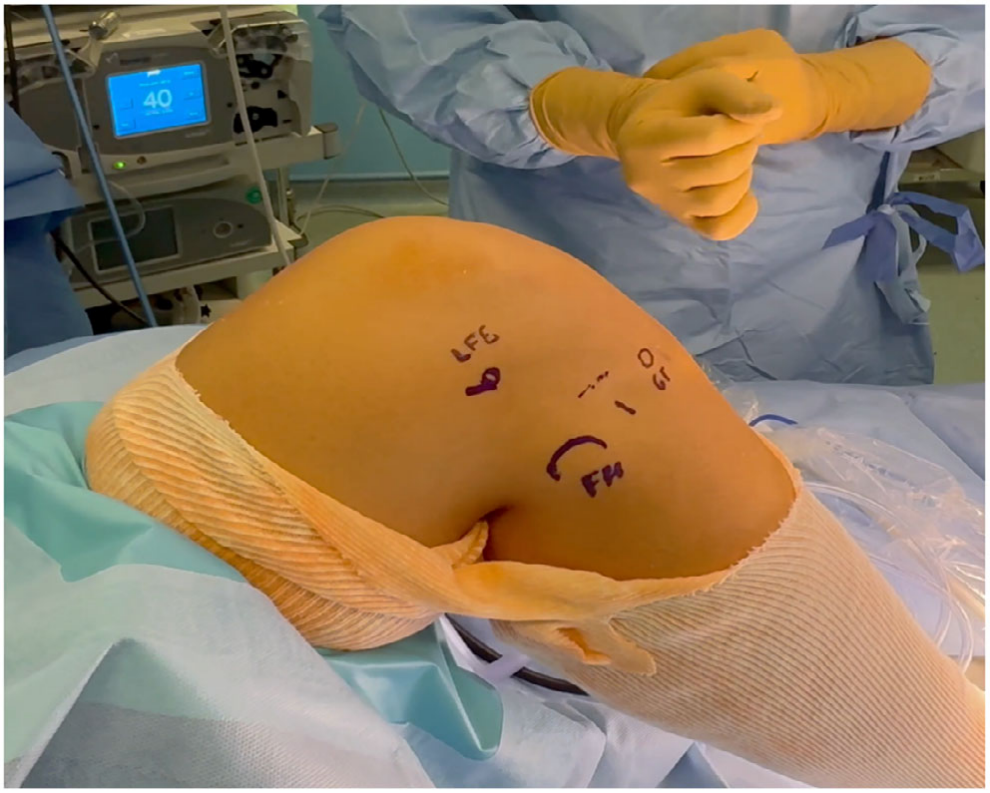
Anatomic landmarks for anterolateral ligament reconstruction. (FH, fibular head; GT, Gerdy's tubercle; LFE, lateral femoral epicondyle.) (Right Knee).

### Hamstring Tendon Harvesting and Precalibration of the Graft

With an open stripper, the tendons of the ST and the gracilis were harvested through a short 3 to 4 cm vertical anteromedial incision.

The ST and gracilis tendons were kept pedicled at their distal insertion to preserve tendon vascularization and improve tibial fixation of the graft (Figure 2).

**FIGURE 2:**
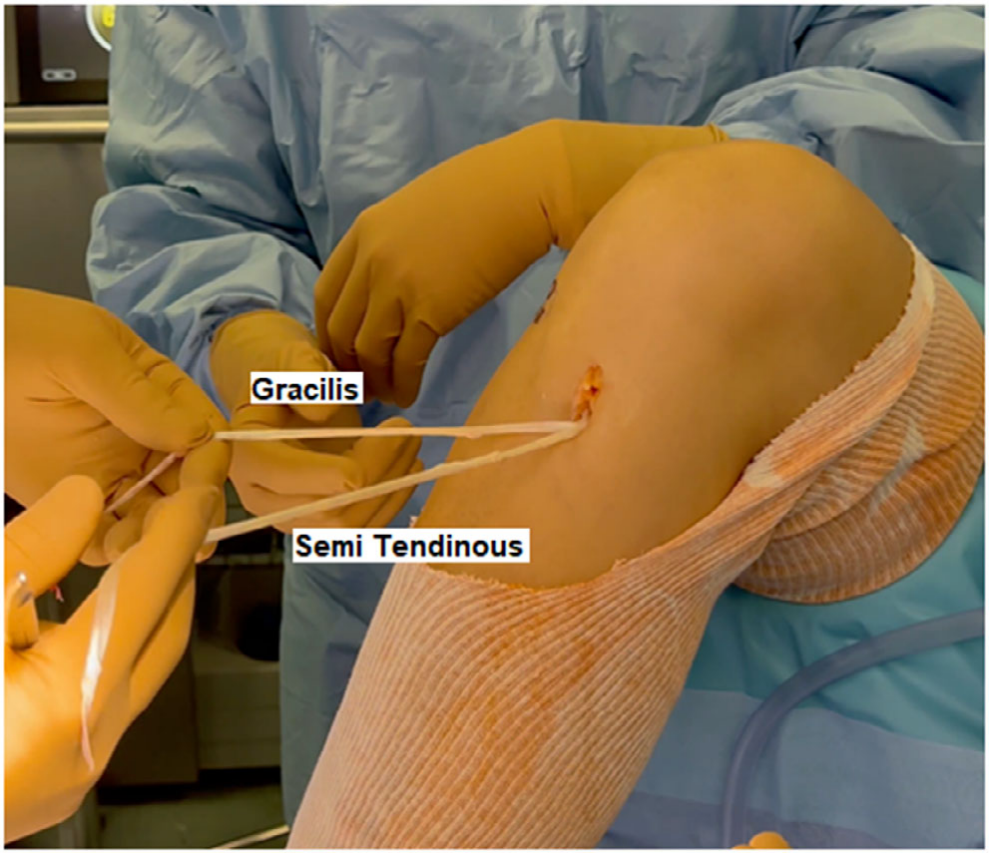
Semitendinosus and gracilis tendons are left pedicled on their tibial insertion. (Right Knee).

The graft was precalibrated to estimate its diameter, which was typically between 8 and 10 mm. This precalibration allowed for the direct preparation of tibial and femoral tunnels with the appropriate diameter.

### ACL Reconstruction

First ACLR was performed in a 4‐strand ST graft kept pedicled.^5^, ^6^

After notch debridement, residual ACL tissue was preserved at its tibial attachment to encourage proprioception, vascularization, and ligamentization of the graft.^7^, ^8^, ^9^, ^10^

An inside‐out guide was used to drill a 25‐mm long femoral tunnel in a diameter that matches that of the graft.^11^ Then, a complete tibial tunnel was drilled from the hamstring incision with an outside‐in guide aimed at the center of the ACL remnant.

This distance was measured using FiberStick (Arthrex, France) from tibial insertion of the ST to the proximal femoral socket. The stick was passed from distal to proximal through the tibial and femoral tunnels. The proximal tip of the stick was fixed at the proximal margin of the femoral socket, while the forceps held it at level of tibial insertion of the ST graft. This was prepared to match the measured distance, corresponding to proper tension.

A mark was made 4 cm from the tibial insertion to ensure that graft length is not less than 10 cm. The graft was tripled over an ACL TightRope RT implant (Arthrex, France), and the looped graft was sutured to itself with FiberLoop (Arthrex, France).

The TightRope, along with ACL graft, was mounted through the tibial tunnel, the ACL remnant, and the femoral tunnel and then secured in place. The interference screw of a diameter equal to the graft was used for tibial fixation at 30° of knee flexion. Finally, the graft was checked under arthroscopy for proper tension and free from interference from the notch in full extension.

### ALL Reconstruction

Two vertical lateral skin incisions were first made. The first was approximately 5 mm proximal and posterior to the lateral epicondyle of the femur, and the second was equidistant between Gerdy's tubercle and the head of the fibula. An overview of ALL reconstruction is described in Video 1.^12^

At the proximal incision, the iliotibial band (ITB) was incised proximally and posteriorly to the lateral epicondyle.

Using a 1.9 mm knotless FiberTak soft anchor (Arthrex, France), a 2.5 mm × 25 mm tunnel was drilled through the cannula into the femoral cortex; while holding the cannula in the same position, the anchor was introduced into the cannula and then impacted into the tunnel. The cannula was removed and firm traction applied to the anchor strands to ensure satisfactory fixation (Figure 3).

**FIGURE 3:**
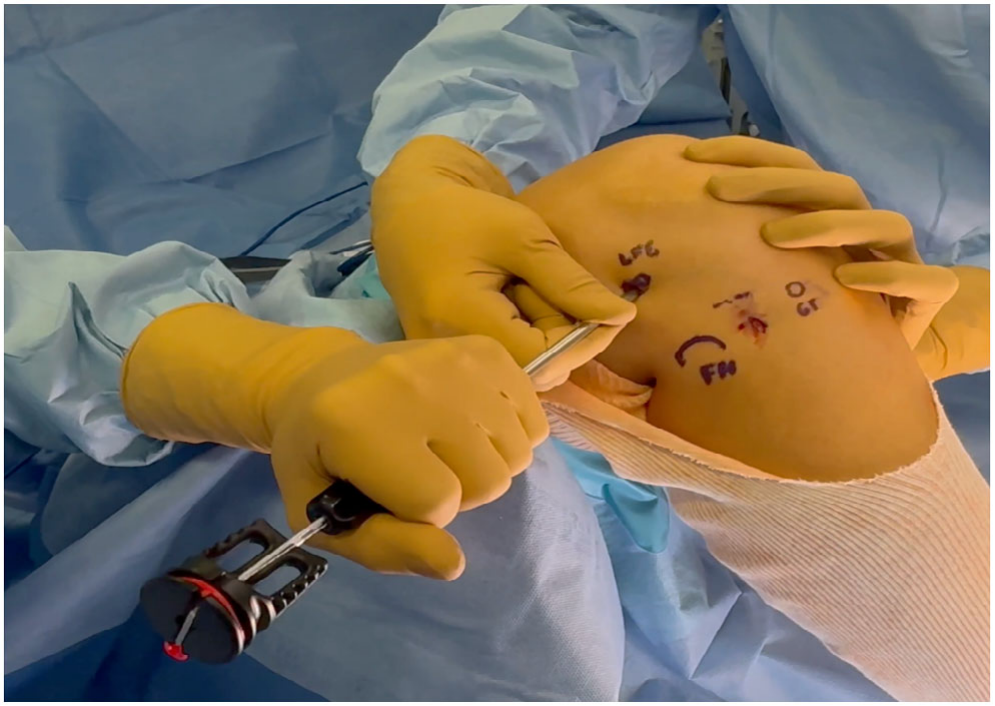
Anchor fixation after drilling tunnel through cannula into femoral bone. (FH, fibular head; GT, Gerdy's tubercle; LFE, lateral femoral epicondyle.) (Right Knee).

Then, entry point was located at midpoint of a horizontal line between Gerdy's tubercle and the head of the fibula, approximately 1 cm from the joint line. The guidewire was passed through and exited at the pre‐existing anteromedial skin incision, just below the ACL tibial tunnel. This step can be performed freehand or with the assistance of the ACL tibial guide to minimize the risk of tunnel convergence.

A 6 to 7 mm‐diameter cannulated drill bit was used to create a complete tunnel (6 mm for women, 7 mm for men) (Figure 4).

**FIGURE 4:**
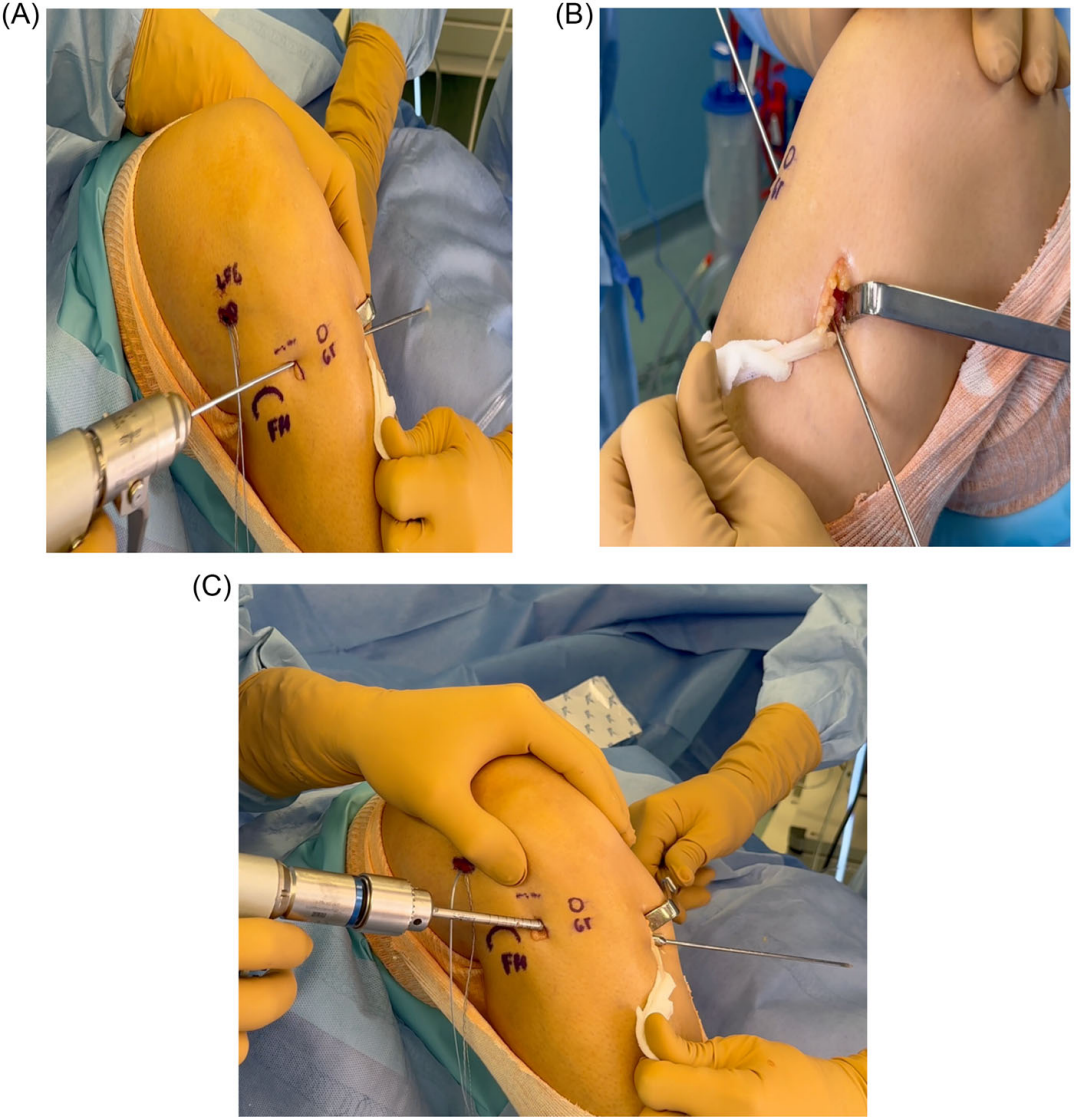
(A) Guidewire passage to prepare tibial tunnel of ALL reconstruction. (B) Exits point through the pre‐existing incision at the graft‐harvesting site on the anteromedial skin. (C) Drilling of a complete tunnel tibial of ALL reconstruction. (ALL, anterolateral ligament; FH, fibular head; GT, Gerdy's tubercle; LFE, lateral femoral epicondyle.) (Right Knee).

The gracilis was prepared and threaded with FiberLoop (Arthrex, France) and passed through the tunnel (Figure 5A).

**FIGURE 5:**
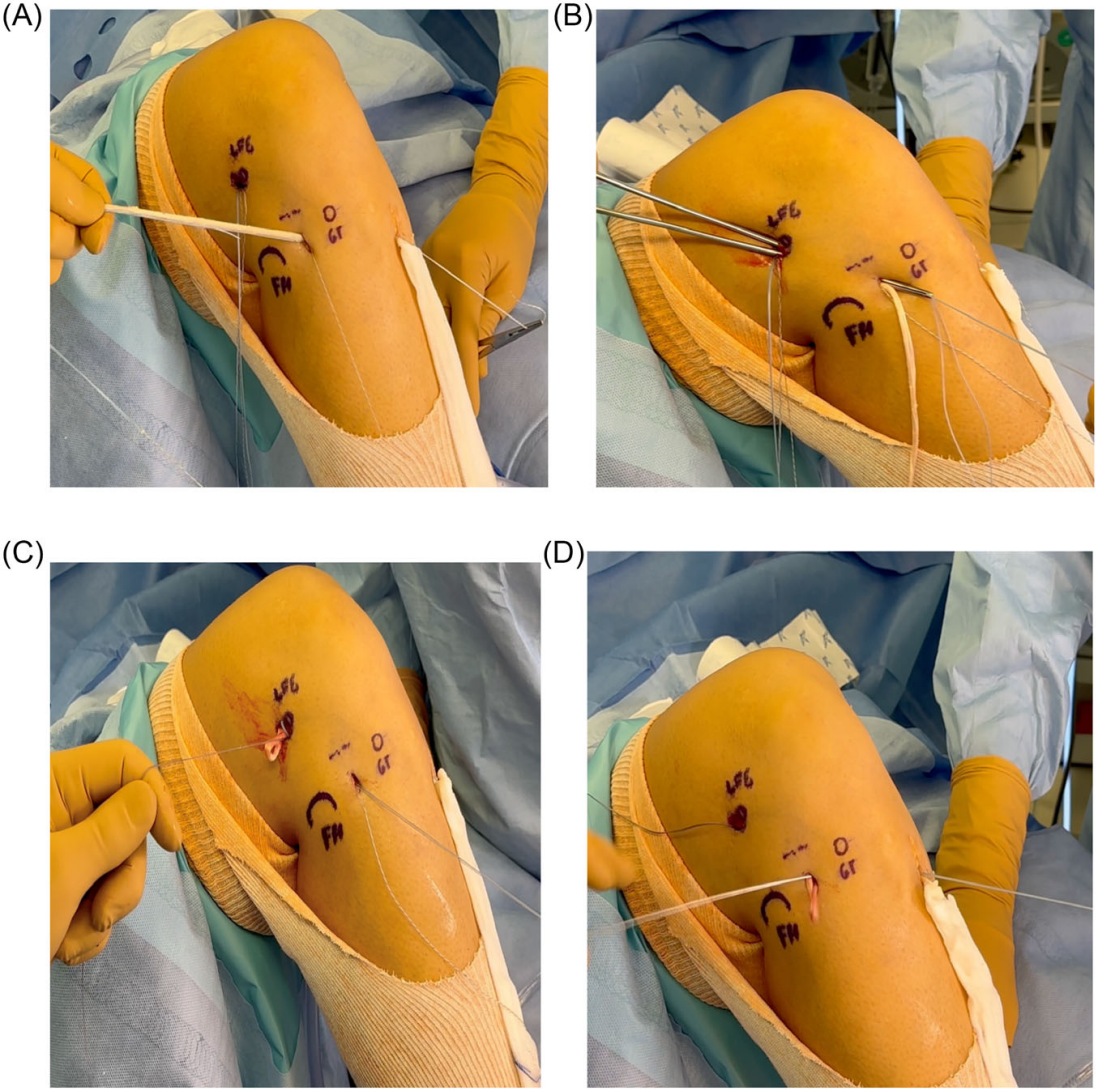
(A) Passage of the gracilis graft through the tibial tunnel. (B) The gracilis tendon is held with forceps and passed from distal to proximal. (C) Femoral fixation of ALL graft by soft anchor. (D) Free end of the graft reintroduced and passed through tibial tunnel. (ALL, anterolateral ligament; FH, fibular head; GT, Gerdy's tubercle; LFE, lateral femoral epicondyle.) (Right Knee).

A Kelly forceps was introduced through the proximal incision under the ITB and superficial to the lateral collateral ligament. The gracilis tendon was pulled with the forceps from distal to proximal, and the anchor fixation thread is passed through the loop of the ALL graft.

The fixation suture was then pulled to secure the proximal part of the graft to the femoral cortex (Figure 5B,C).

Subsequently, the free end of the gracilis tendon was reintroduced from proximal to distal and then passed through the tibial tunnel (Figure 5D).

The graft was secured in the tibial tunnel using a 6 or 7 mm interference screw (same than tunnel diameter), with the knee in full extension and neutral rotation (Figure 6).

**FIGURE 6:**
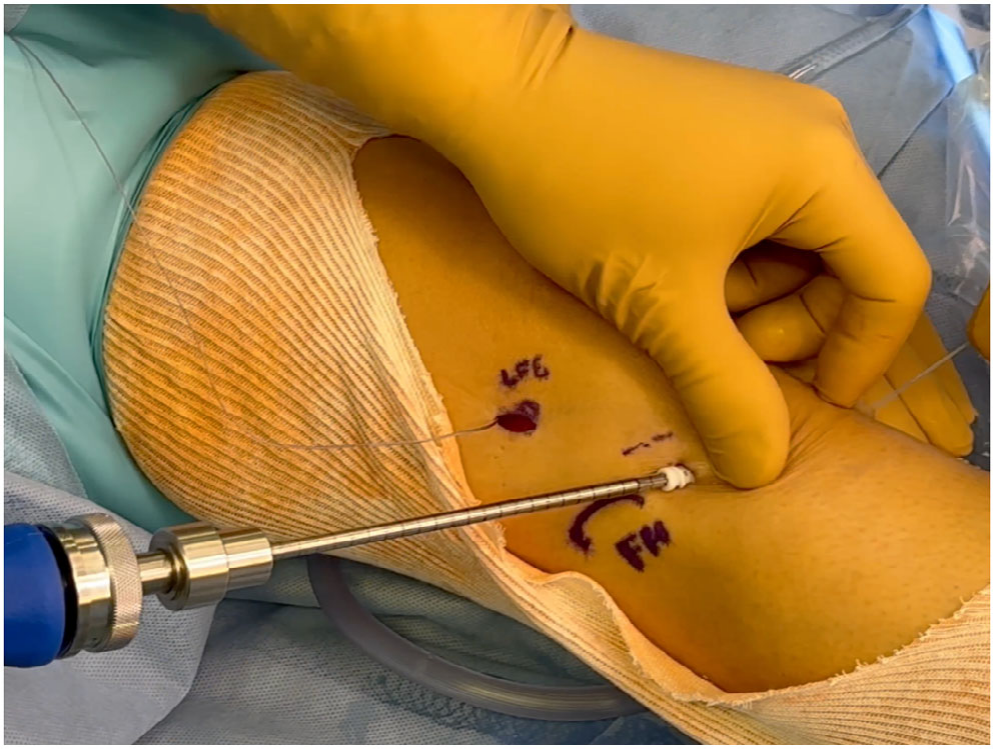
Tibial fixation of ALL graft with interference screw in full extension and neutral rotation of the knee. (ALL, anterolateral ligament; FH, fibular head; LFE, lateral femoral epicondyle.) (Right Knee).

Tips and pearls of the surgical technique are listed in Table 1.

**TABLE 1:** Tips and Pearls of This Surgical Technique

Tips and pearls of the surgical technique
• Tendon grafts are soaked in a vancomycin solution
• ACL graft is passed into the tunnels only after drilling the ALL tunnel to ensure there is no convergence between femoral tunnels
• A soft anchor is used as it directly applied on the cortex and penetrates less deeply into the bone
• This reduces reduce the risk of tunnel convergence
• There is no need to measure graft length, because the gracilis is consistently long
• Tibial tunnel diameter should be 6 mm for women and 7 mm for men
• ALL fixation is performed after ACL fixation, with knee in full extension and neutral rotation.

ACL, anterior cruciate ligament; ALL, anterolateral ligament.

### Postoperative Rehabilitation

All patients undergoing ACL reconstruction are treated on an outpatient basis. A cryotherapy brace is applied for the first 2 days to manage swelling and pain. Rehabilitation is started on the first postoperative day, with full weight‐bearing allowed while using crutches for 2 weeks. The rehabilitation program follows three phases: recovery, strength and mobility, and return to sport.

## DISCUSSION

This technique describes an anatomical reconstruction of the ALL using a pedicled gracilis graft, performed independently of ACL reconstruction. It is a simple, reproducible, and minimally invasive method that respects anatomical landmarks, preserves the native tibial attachment of the graft, offering biological advantage that promote vascularized healing and natural graft incorporation, and effectively restores rotational knee stability.

Combined ACL and ALL injuries are common, occurring in 46% to 79% of cases according to the literature,^13^, ^14^, ^15^ which justifies the interest in combined reconstruction to effectively restore knee rotatory stability. Furthermore, combined ACL and ALL reconstruction is associated with a significant reduction in the graft failure rate and better preservation of medial meniscus repairs.^16^, ^17^, ^18^, ^19^ Among the various extra‐articular plasty techniques, it would appear that anatomic reconstruction of the ALL is preferable to the various extra‐articular lateral tenodesis techniques. Indeed, the latter could lead to a limitation of joint kinematics, excessive tension on the lateral compartment, thus favoring the development of degenerative osteoarthritis and, consequently, less satisfactory clinical results.^20^, ^21^

On a biological point of view, preserving the tibial insertion of the gracilis tendon maintains its native vascular supply, which promotes biological integration, healing, and incorporation of the graft into the bone tunnels. This biological principle is in line with the ST3+2 technique described by Sonnery‐Cottet for ACL reconstruction, where the graft remains dependent and continuously vascularized.^5^, ^6^, ^22^, ^23^ This approach enhances graft viability and strengthens tibial fixation.

This procedure also has minimal impact on femoral bone stock, only a 2.5 to 25‐mm tunnel is needed to impact the anchor into the femoral cortex. A similar use of a soft anchor for femoral fixation was reported by Mesnier et al.^24^ It has been shown that cortical attachment provides satisfactory graft incorporation. Additionally, it prevents ACL and ALL tunnel convergence, which can occur in 67% of cases and becomes a major issue when weak femoral attachment compromises ACL reconstruction efficacy.^25^

This approach involves two small skin incisions and follows a standardized, anatomical trajectory for the graft. A complete tibial tunnel is drilled with a fixed diameter of 6 for female patients or 7 mm for males, ensuring that the graft always passes easily and eliminating variability. The graft length is consistently sufficient due to the preserved tibial attachment. This makes the technique highly reproducible and easily teachable, with minimal dissection and no need to harvest the ITB as required in other extra‐articular techniques or complex measurements.^6^, ^16^, ^17^, ^26^ Our method offers an alternative by using an independent ALL graft, suitable for surgeons preferring in‐out femoral fixation.

Nevertheless, our technique is limited by the requirement of a complete tibial tunnel for ALL. When an out‐in femoral technique is planned, a continuous graft configuration may be more suitable. The procedure also involves a specific anchoring implant, which may represent an additional cost. It also necessitates the combined use of the ST and gracilis tendon (Table 2).

**TABLE 2:** Advantages and Limitations of This Surgical Technique

Advantages
• Preservation of ACL remnant to improve ligamentizatio
• ACL and ALL graft are pedicled to tibia to encourage vascularization
• ALL and ACL graft techniques are independent
• The technique is less invasive, and there is no need for ITB harvesting compared with popular LET techniques
• There is no need to measure tunnel lengths for ALL
• Nonmetallic fixation

Limitations
• Both semitendinosus and gracilis grafts are needed
• Additional implant cost
• Complete tibial tunnel for ALL

ACL, anterior cruciate ligament; ALL, anterolateral ligament; ITB, iliotibial band; LET, lateral extra‐articular tenodesis.

Finally, it is an easily reproducible technique that does not increase comorbidity. What is more, it uses a vascularized tendon, which promotes integration. Further studies are needed to assess the long‐term clinical results of this technique and compare them with other approaches.

In conclusion, this technique offers a reproducible and minimally invasive method to anatomically reconstruct the ALL using a pedicled graft that maintains its native tibial attachment. It avoids femoral tunnel convergence, supports biological integration, and serves as a valuable option for combined reconstructions of the ACL and ALL. Its simplicity, safety profile, and alignment with contemporary anatomical and biomechanical standards make it a compelling alternative for surgeons managing rotatory knee instability.

## DISCLOSURES

The authors (I.M., T.N.) declare the following financial interests/personal relationships which may be considered as potential competing interests: I.M. reports financial support was provided by Arthrex. T.N. reports financial support was provided by Arthrex. The other author (A.V.) declares that they have no known competing financial interests or personal relationships that could have appeared to influence the work reported in this article.
